# Lipid regulation of adenylyl cyclase Rv1625c from *Mycobacterium tuberculosis* by its membrane‐domain receptor

**DOI:** 10.1111/febs.70148

**Published:** 2025-06-01

**Authors:** Anita Charlotte Friderun Schultz, Marius Landau, Andrei N. Lupas, Joachim Erdmann Schultz

**Affiliations:** ^1^ Max‐Planck‐Institute für Biologie Tübingen Germany; ^2^ Pharmazeutisches Institut Universität Tübingen Germany

**Keywords:** adenylyl cyclase, lipid receptor, mycobacterium, oleic acid, regulation

## Abstract

The regulation of mammalian adenylyl cyclases by G‐protein‐coupled receptors and the Gsα subunit of trimeric G‐proteins has been extensively studied, whereas little is known about the regulation of their closely related bacterial cyclases. Here, we focused on the regulation of the adenylyl cyclase Rv1625c from *Mycobacterium tuberculosis H37Rv*. Rv1625c is a progenitor of mammalian congeners. Exclusively C_18_‐mono‐unsaturated fatty acids, such as the cis‐ and trans‐isoforms of oleic and vaccenic acids, inhibited the Rv1625c holoenzyme with IC_50_ concentrations around 10 μm. The saturated C_18_ fatty acid stearic acid was inactive. A soluble Rv1625c construct, which lacked the membrane domain, was not affected by the mono‐unsaturated C_18_ fatty acids, i.e., the inhibition required the presence of the membrane domain, indicating a receptor–ligand interaction. Surprisingly, fatty acid inhibition of Rv1625c was strictly dependent on magnesium ions (Mg^2+^) as a divalent cation for the substrate adenosine triphosphate (ATP). Although manganese ion (Mn^2+^)–ATP as a substrate greatly increased enzyme activity, Mn^2+^ appeared to block intramolecular signal transduction from the membranous receptor domain to the catalytic effector domain. In summary, the results bolster the proposal that adenylyl cyclase regulation by fatty acids is an evolutionarily conserved signaling mode present in bacteria as well as in mammals.

## Graphical abstract

The adenylyl cyclase (AC) Rv1625c from *M. tuberculosis* is a monomeric progenitor of the nine pseudoheterodimeric mammalian AC isoforms. The hexahelical membrane anchor of Rv1625c serves as an inhibitory receptor for fatty acids, particularly oleic acid. One ligand binds into each monomer of the active Rv1625c dimer. This lipid regulation is similar to the inhibition of mammalian AC isoforms 1, 4, 5, and 6 by aliphatic lipids.

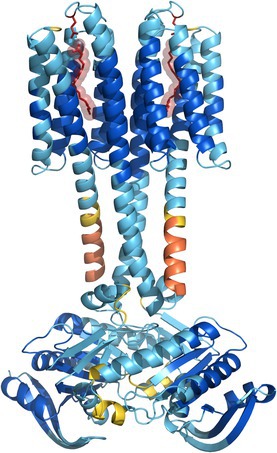

**Abbreviations**
AC: adenylyl cyclase
cryoEM: cryo‐electron‐microscopy
GPCR: G‐protein‐coupled receptor
mAC: membrane‐delimited adenylyl cyclase
TM: membrane domain

## Introduction

Adenosine 3′,5′‐monophosphate (cAMP) is a nearly universal second messenger, already present in cyanobacteria, one of the earliest forms of life [1, 2, 3, 4]. The synthesis of cAMP is catalyzed by adenylyl cyclases (ACs), which use ATP as a substrate and either Mn^2+^ or Mg^2+^ as divalent cations. To date, six distinct classes of ACs have been identified, with class III being by far the most diverse. Class III ACs are present in prokaryotes as well as in eukaryotes [2]. Bacterial class III ACs are typically monomers in which the N‐terminus mostly is preceded by distinct regulatory domains [5]. For catalytic activity, dimerization is essential, as the catalytic center forms at the dimer interface [1, 2, 6]. In bacterial ACs a variety of N‐terminal regulatory domains have been identified [2, 4, 5]. In contrast, the mammalian membrane‐delimited ACs (mACs) are pseudoheterodimers, comprising a hexahelical membrane domain (TM1), a catalytic domain (C1), a second membrane domain (TM2), and a second catalytic domain (C2). Probably, the mammalian ACs evolved through concatenation of a monomeric bacterial progenitor, such as the mycobacterial mAC Rv1625c from *Mycobacterium tuberculosis H37Rv* [1].

We recently reported that the dodekahelical membrane domain of mammalian ACs is a receptor for aliphatic lipids, such as fatty acids or anandamide [7]. At low micromolar concentrations, the ligands modulate the extent of Gsα stimulation, either enhancing (AC isoforms 2, 3, 7, and 9) or attenuating (isoforms 1, 4, 5, and 6) Gsα activation, *in vitro* and *in vivo*. In bacteria, G‐protein‐coupled receptors and trimeric G‐proteins are absent, raising questions about the regulation of bacterial ACs. In *M. tuberculosis*, 16 ACs have been predicted [8] and in the 12 sequenced *Mycobacteria* strains a total of 170 class III ACs have been identified (https://www.ncbi.nlm.nih.gov/Complete_Genomes/SignalCensus.html; range 4–31) [9]. In this study, we focused on a possible regulatory function of the hexahelical membrane domain of the AC Rv1625c from *M. tuberculosis H37Rv*, which has many mycobacterial homologs [9]. We asked whether Rv1625c activity might be similarly regulated by aliphatic fatty acids like its eukaryotic congeners. We show that mono‐unsaturated C‐18 fatty acids inhibit Rv1625c activity via its anchor‐receptor. Remarkably, inhibition required the use of Mg^2+^‐ATP as a substrate. No ligand effect was observed with Mn^2+^‐ATP. The results raise intriguing questions about the evolution and regulation of class III ACs in both pro‐ and eukaryotes.

## Results

**Fig. 1:**
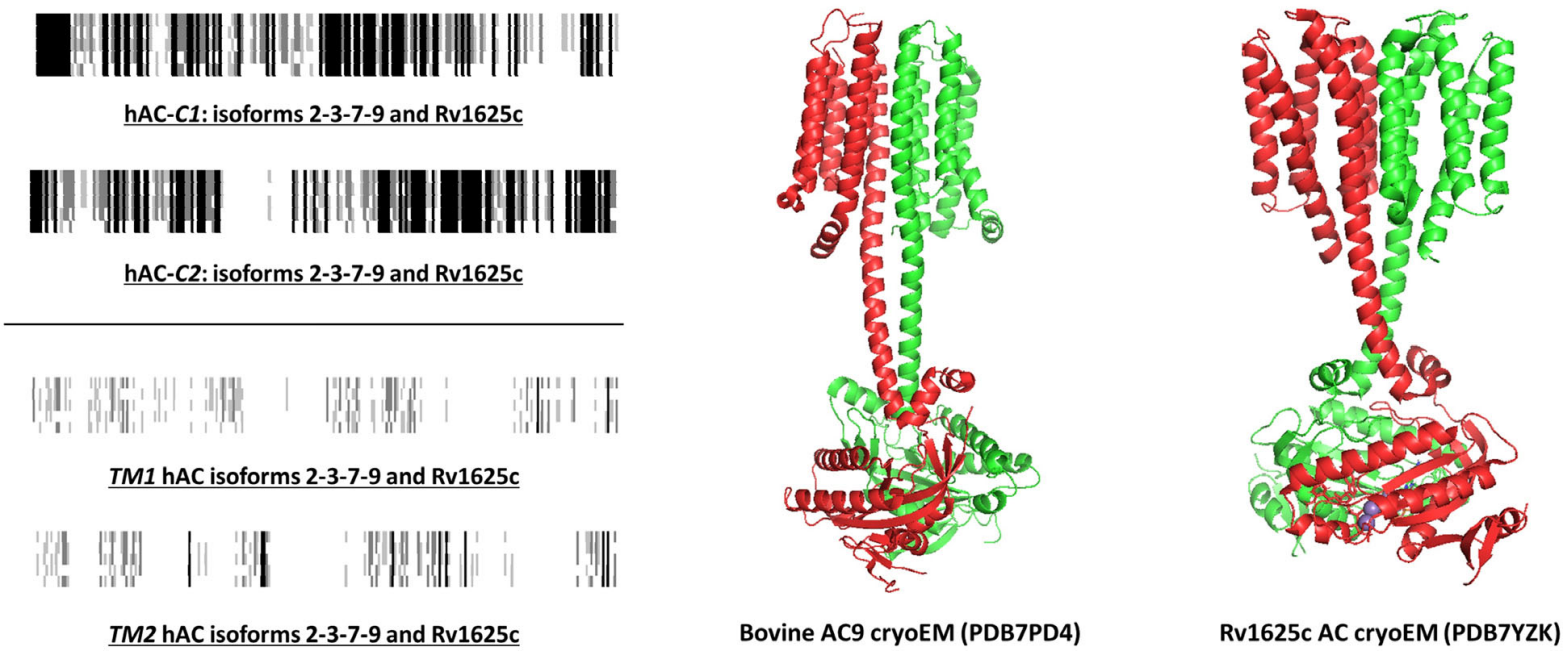
Sequence alignments of pseudoheterodimeric mammalian adenylyl cyclases with the monomeric mycobacterial adenylyl cyclase Rv1625c and corresponding structures. Left: alignments of the catalytic and transmembrane domains of Rv1625c with the equivalent domains from human adenylyl cyclase isoforms 2, 3, 7, and 9 (hAC‐C1, first catalytic domain; hAC‐C2, second catalytic domain; TM1 hAC, transmembrane domain 1 from human adenylyl cyclases 2, 3, 7, and 9; TM2 hAC, transmembrane domain 2 from human adenylyl cyclases 2, 3, 7, and 9 (generated with genedoc). Right: comparison of structures determined by cryo‐electron microscopy (cryoEM) of the bovine adenylyl cyclase isoform 9 (pdb 7PD4) and the homodimer of the mycobacterial Rv1625c (pdb 7YZK) [9, 13, 14]. The amino acid boundaries used for the alignments are listed in Table 1. Shading in alignments: black, invariant; dark gray, conserved; light gray, slightly conserved; white: disparate.

The catalytic domains of all class III ACs are highly conserved. In contrast, the N‐terminal domains are highly diverged [2, 10]. We aligned the single catalytic domain of the mycobacterial mAC Rv1625c individually with the C1 and C2 catalytic domains of the human mAC isoforms 2, 3, 7, and 9 (Fig. 1, top left). In the latter isoforms, the mono‐unsaturated fatty acid oleic acid significantly enhances Gsα‐stimulated enzyme activity [7]. Obviously, the catalytic domains display a high degree of identity and similarity (Fig. 1, top left). In contrast, the alignment of the single hexahelical membrane domain of Rv1625c with either the membrane domain TM1 or TM2 of the human mACs 2, 3, 7, and 9 reveals little conservation (Fig. 1, bottom left). Yet, the available cryoEM structure of the mammalian mAC isoform 9 is very similar to that of the active Rv1625 dimer (Fig. 1, right).

**Table 1:** The amino acid boundaries used for the alignments in Fig. 1.

	Cat. C1_2–3–7–9 Rv1625c		Cat. C2_2–3–7–9 Rv1625c		TM1 hAC2–3–7–9 Rv1625c		TM2 hAC2–3–7–9 Rv1625c	
AC‐2	S290	P473	V888	T1078	L46	Y207	V604	G821
AC‐3	S319	T502	V924	G1121	L78	M262	F636	F857
AC‐7	S279	P462	V880	T1069	L34	F196	F597	S814
AC‐9	S394	C581	V1059	K1244	Y118	H297	L793	N997
Rv1625c	S251	G431	V252	G423	V47	V198	V47	W199

The structure of the Rv1625c mAC dimer has been elucidated by X‐ray crystallography and cryo‐EM [9, 11, 12]. The two membrane domains combine into a dodekahelical complex, essentially identical to that observed in the available cryo‐EM structures of the pseudoheterodimeric mAC isoforms 5, 8, and 9 [9, 11, 13, 14]. This suggests that the dimeric Rv1625c membrane complex might serve as a ligand receptor as do the membrane domains of mammalian mACs (see Fig. 1, right; [14, 15]).

The activities of bacterial ACs generally are rather low, either due to low expression or to an intrinsically low activity. This might be due to the tiny volume of bacteria, e.g., approximately 800 attoliter for *E. coli* or 300 attoliter for *M. tuberculosis*. Thus, only 600 or 225 cAMP molecules per cell are needed to reach a 1 μm concentration. Experimentally this issue is usually bypassed by using Mn^2+^‐ATP as a substrate, which multiplies AC activities compared to Mg^2+^‐ATP. In our experiments, the activity of Rv1625c with 2 mm Mn^2+^‐ATP as a substrate was 2 μmol cAMP·mg^−1^·min^−1^ compared to 0.24 with 2 mm Mg^2+^‐ATP, an eightfold difference. Similar results were observed with a soluble construct, Rv1625c_CAT‐(203–443)_2_. The activity with Mn^2+^‐ATP was 8.65 compared to 0.25 nmol cAMP·mg^−1^·min^−1^ with Mg^2+^‐ATP, reflecting a 34‐fold difference. Therefore, we used Mn^2+^‐ATP as a substrate to leverage the higher basal activity [6, 16, 17, 18].

**Fig. 2:**
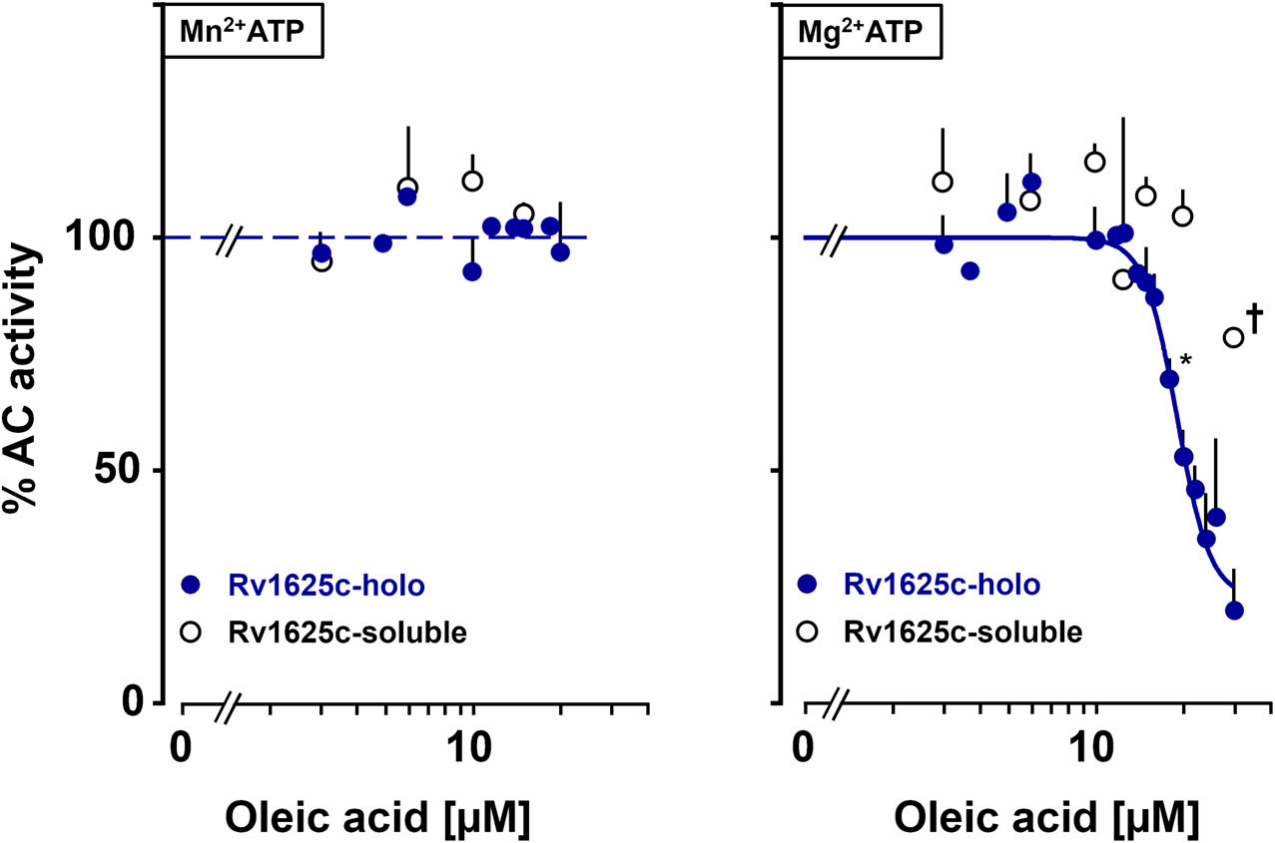
Effect of oleic acid on Rv1625c holoenzyme and the soluble dimer Rv1625c‐CAT(204–443)_2_. Left: with 2 mm Mn^2+^‐ATP as a substrate; right: with 2 mm Mg^2+^‐ATP as a substrate. Activities are depicted as % compared to respective basal activities (= 100%). Basal activities of the soluble dimer with Mg^2+^ and Mn^2+^‐ATP were 0.2 ± 0.01 and 8.8 ± 2.7 nmol cAMP·mg^−1^·min^−1^ (*n* = 3). Basal activities of the Rv1625c holoenzyme with Mg^2+^ or Mn^2+^ were 0.31 ± 0.05 and 2.0 ± 0.29 μmol cAMP·mg^−1^·min^−1^, respectively (*n* = 4). The IC_50_ for oleic acid was 18 μm. Error bars denote SEM Significances (one sample *t*‐test; graphpad 10.4.1): *P < 0.01 compared to 100%: ^†^ *P* < 0.01 compared to the respective Rv1625c holoenzyme point (unpaired *t*‐test).

As an initial ligand probe we used the mono‐unsaturated C18 carboxylic acid oleic acid for three reasons: (a) in the structure of the dimer of the cytosolic mycobacterial AC Rv1264 oleic acid was found to be tightly packed between the regulatory and catalytic domains [19, 20]; (b) in the mycobacterial AC Rv2212, related to Rv1264, > 50 μm oleic acid activated up to eight‐fold [21]; (c) oleic acid greatly enhanced enzyme activity in the Gsα‐stimulated activities of the human mAC isoforms 2, 3, 7, and 9 [7]. However, using Mn^2+^‐ATP as a substrate up to 20 μm oleic acid did not affect enzyme activities of the Rv1625c holoenzyme or the soluble construct Rv1625c‐CAT‐(203–443)_2_ (Fig. 2, left). Simultaneously, the data ruled out detrimental surfactant effects of oleic acid.

It is unlikely that *in vivo* Mn^2+^ serves as the divalent cation in the cyclase reaction, as mm concentrations of Mn^2+^ have not been reported in *E*. *coli* or other bacteria [22]. In general, a detailed mechanism of class III AC activation by Mn^2+^ ions has not been reported [23, 24, 25, 26, 27, 28, 29, 30]. We repeated the oleic acid concentration‐response curves using 2 mm Mg^2+^‐ATP and a 10‐fold higher protein concentration in the assays (0.45 ng with Mg^2+^ vs. 0.045 ng with Mn^2+^). Surprisingly, oleic acid strongly inhibited Rv1625c holoenzyme. Inhibition started around 12 μm oleic acid and reached 80% at 30 μm. The IC_50_ for oleic acid was 18 μm (Fig. 2, right). The steep decline over a narrow concentration range prompted us to evaluate the curve for cooperativity. A Hill coefficient of 7.6 strongly indicated positive cooperativity, i.e., we may assume that two or more oleic acid molecules may bind. The soluble construct lacking the membrane domain was unaffected under identical assay conditions (Fig. 2, right). Only at 30 μm, oleic acid caused a 20% decrease in activity, highly significantly different from the corresponding point with the Rv1625c holoenzyme (80% inhibition, *P* < 0.01; Fig. 2, right). The data suggested that oleic acid acted in a membrane receptor‐ligand fashion. This is similar to the lipid‐receptor interactions reported for the mammalian AC isoforms 1, 2, 3, 4, 5, 6, 7, and 9 [7]. Furthermore, we unequivocally determined that Mg^2+^ cations were required for intramolecular signal transduction because, in the presence of Mn^2+^, no inhibition was observed.

**Fig. 3:**
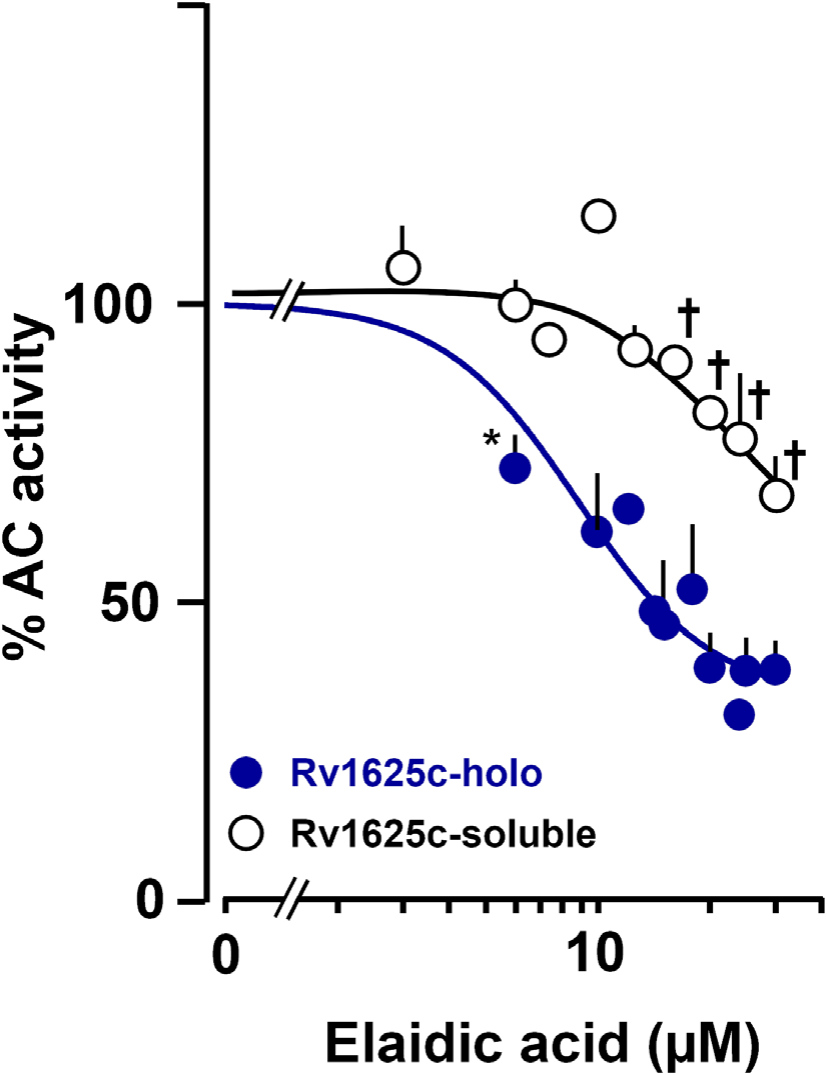
Effect of elaidic acid on Rv1625c holoenzyme and the soluble dimer Rv1625c‐CAT(204–443)_2_. 2 mm Mg^2+^‐ATP was used as a substrate. Basal activities of Rv1625c‐CAT‐(203–443)_2_ and Rv1625c‐mAC were 190.2 ± 37 nmol and 0.2 ± 0.03 nmol cAMP·mg^−1^·min^−1^. Activities are % of respective basal activities. The IC_50_ concentration for elaidic acid was 8.9 μm (*n* = 3). Error bars denote SEM Significances (one sample *t*‐test; graphpad 10.4.1): *P < 0.01 compared to 100%. ^†^ *P* < 0.01 compared to respective Rv1625c holoenzyme points (unpaired *t*‐test).

9‐Octadecenoic acid exists in two conformations: the cis‐isomer (oleic acid; (Z)‐9‐octadecenoic acid) and the trans‐isomer (elaidic acid; (E)‐9‐octadecenoic acid). Elaidic acid inhibited the Rv1625c holoenzyme distinctly differently (Fig. 3). The minimal concentration for inhibition was 6 μm, i.e., it had greater potency compared to oleic acid, but lower efficacy with maximally 60% inhibition at 30 μm. The IC_50_ for elaidic acid was 8.9 μm (Fig. 3). Oleic acid, with its 120‐degree kink at the 9,10 position, is structurally contorted compared to elaidic acid. With a melting point of 14 °C, oleic acid is fluid and highly flexible at the incubation temperature of 37 °C. The melting point of elaidic acid (46 °C) may indicate a somewhat diminished flexibility at the assay temperature. The results may suggest that a critical fluidity of the C18 aliphatic side chain may be important for ligand binding.

**Fig. 4:**
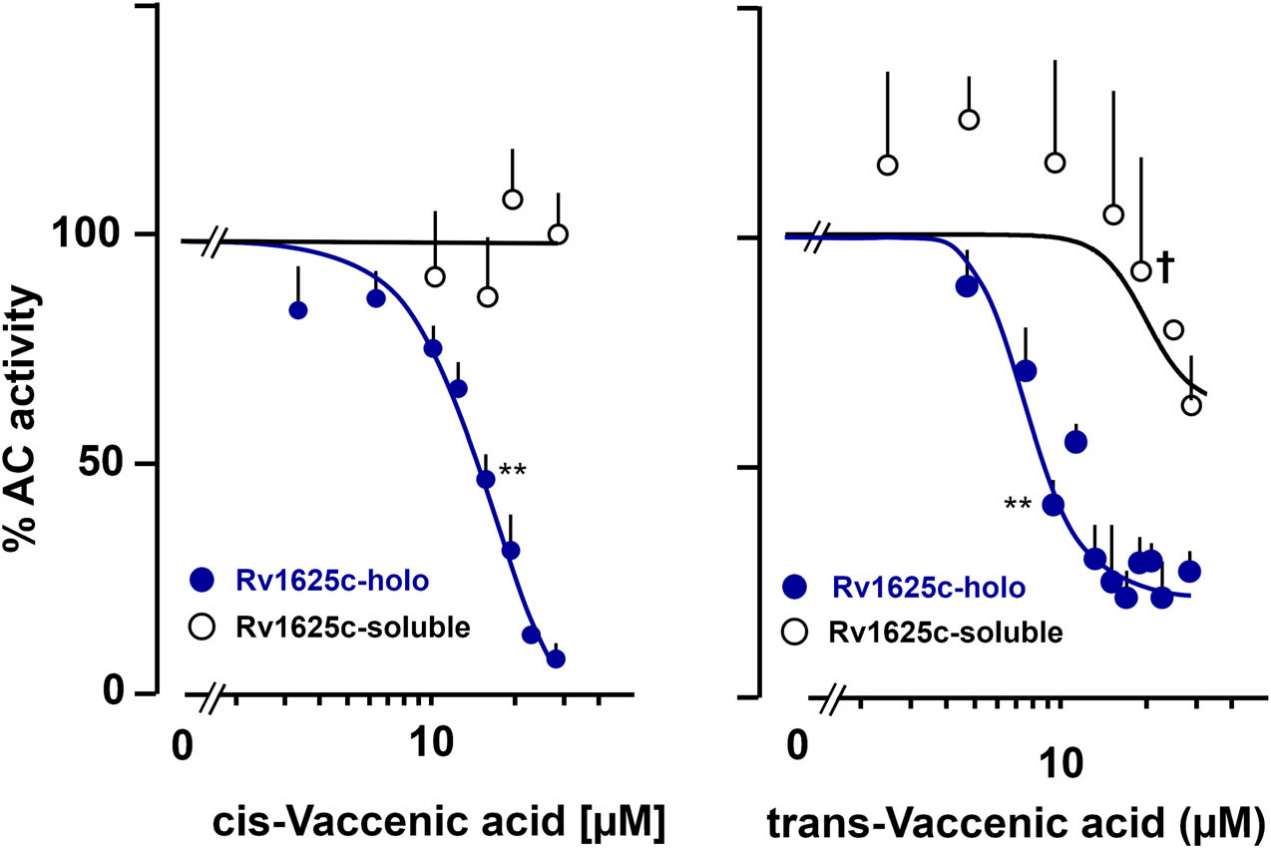
Effect of *cis*‐ and *trans*‐vaccenic acids on Rv1625c and soluble Rv1625c‐CAT‐(203–443)_2_ using 2 mm Mg^2+^‐ATP as a substrate. Left: *cis*‐Vaccenic acid. The IC_50_ is 17.7 μm. Activities are in % of respective basal activities (100%). Basal activity of Rv1625c and Rv1625c soluble construct were 135.6 ± 42 and 0.38 ± 0.14 nmol cAMP·mg^−1^·min^−1^, respectively. Error bars denote SEM Significances (one sample *t*‐test; (graphpad 10.4.1): **P < 0.01 compared to 100%. (*n* = 2). Right: *trans‐*Vaccenic acid. Basal activities of Rv1625c and soluble Rv1625c construct were 50.9 ± 5.8 and 0.27 ± 0.06 nmol cAMP·mg^−1^·min^−1^, respectively. Activities are in % of respective basal activities (100%). IC_50_: 8.9 μm. Error bars denote SEM (*n* = 2). Significances (one sample *t*‐test; graphpad 10.4.1): **P < 0.01 compared to 100%. ^†^ *P* < 0.01 compared to respective Rv1625c holoenzyme points (unpaired *t*‐test).

Another biologically important mono‐unsaturated C18 carboxylic acid is vaccenic acid, (E)‐11‐ and (Z)‐11‐octadecenoic acids. Concentration‐response curves for cis‐ and trans‐conformations presented characteristic differences (Fig. 4). Cis‐vaccenic acid inhibited the Rv1625c holoenzyme with identical efficacy (> 90% inhibition at 30 μm) and potency (IC_50_ = 18 μm) as did (E)‐9‐octadecenoic acid (oleic acid). It did not affect the soluble construct. In contrast, trans‐vaccenic acid showed higher inhibitory potency (IC_50_ = 8.9 μm), but consistently lower efficacy (75% inhibition at 30 μm; Fig. 4), like elaidic acid. The melting points for the cis‐ (14 °C) and trans‐ (44 °C) isomers differed similarly as the isomer couple oleic and elaidic acid. The results further support the notion that fatty acid flexibility is an important parameter for ligand binding.

**Fig. 5:**
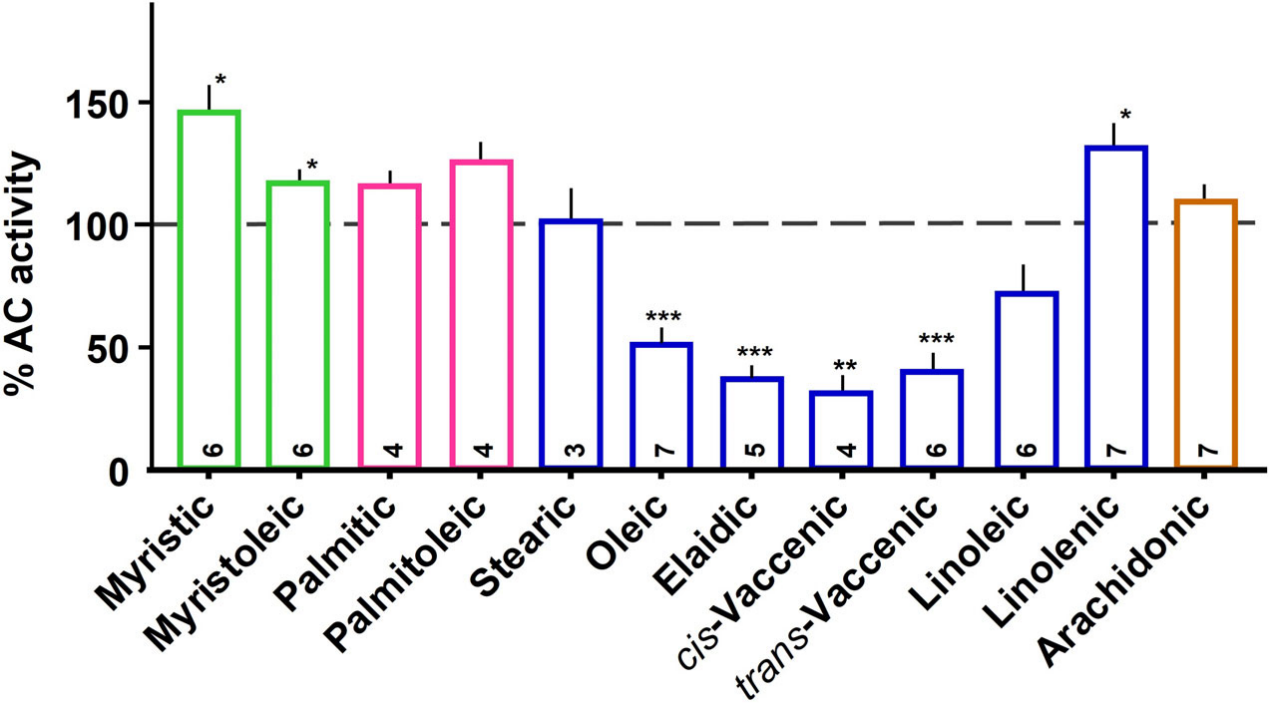
Effect of 20 μm lipids on Rv1625c adenylyl cyclase activity. 2 mm Mg^2+^‐ATP was used as a substrate. Activities are shown in % compared to respective basal activities. The number of independent experiments (each with two technical repeats) is indicated at the bottom of the bars. Values are means ± SEM Significances (one sample *t*‐test; graphpad 10.4.1): *P < 0.05; **P < 0.01; ***P < 0.001 compared to 100%. Green bars: C14–fatty acids; pink bars: C16–fatty acids; blue bars: C18–fatty acids; brown bar: C_20_, arachidonic acid.

Further exploration of the ligand space revealed that 20 μm of the saturated C18 carboxylic acid stearic acid did not inhibit (Fig. 5). Higher unsaturated fatty acids, such as linoleic acid, (9*Z*,12*Z*)‐octadeca‐9,12‐dienoic acid, α‐linolenic acid, (9*Z*,12*Z*,15*Z*)‐octadecatrienoic acid, and arachidonic acid, (all‐Z)‐5,8,11,14‐eicosatetraenoic acid, were ineffective (Fig. 5). The melting points of the latter fatty acids are below 0 °C. Probably the steric restrictions due to multiple double bonds impair the binding of the fatty acid into a tight binding pocket. The shorter C16 fatty acids palmitic acid (saturated) and mono‐unsaturated palmitoleic acid ((9Z)‐hexadecenoic) could not serve as ligands. We also tested methyl oleate and *N*‐oleoyl glycine, i.e., compounds in which the carboxyl group was derivatized. Neither compound affected Rv1625c holoenzyme activity up to 30 μm indicating that the carboxyl group probably was required for ligand functionality.

In summary then, the data indicate that C‐18 mono‐unsaturated aliphatic fatty acids with a free carboxyl group inhibited the activity of the Rv1625c mAC holoenzyme within a rather narrow concentration range, whereas the soluble domain was barely affected under identical conditions.

## Discussion

Recently, we reported that the membrane domains of the mammalian AC isoforms serve as lipid receptors [7]. The ligands directly control the extent of the stimulation via GPCRs and Gsα. The bacterial mAC Rv1625c has a domain order reflecting exactly one half of the pseudoheterodimeric mammalian ACs (Fig. 1; [1]). Consequently, this study was restricted to the question of a potential receptor function of the membrane domain of Rv1625c. The direct lipid regulation of Rv1625c probably defines a primordial signaling system, predating by far the subsequent evolution of the indirect regulation via GPCRs. The results highlight four fundamental aspects of class III AC regulation, which are discussed in turn below.

First, this is the first example of direct lipid signaling in a bacterial class III mAC. The physically linked, soluble dimer Rv1625c‐CAT‐(203–443)_2_, lacking the membrane domains, is unaffected, i.e., oleic acid inhibits Rv1625c via binding to theRv1625c membrane complex and the dimer of the Rv1625c membrane domains is identified as a lipid receptor [7]. As a lipid receptor composed of 12 α‐helices, it differs distinctly from previously identified lipid and fatty acid receptors in eukaryotic cells, which are canonical heptahelical G‐protein‐coupled receptors [31, 32, 33]. In fact, the data appear to establish the existence of a new mode of lipid signaling clearly distinct from lipid signaling via GPCRs.

**Fig. 6:**
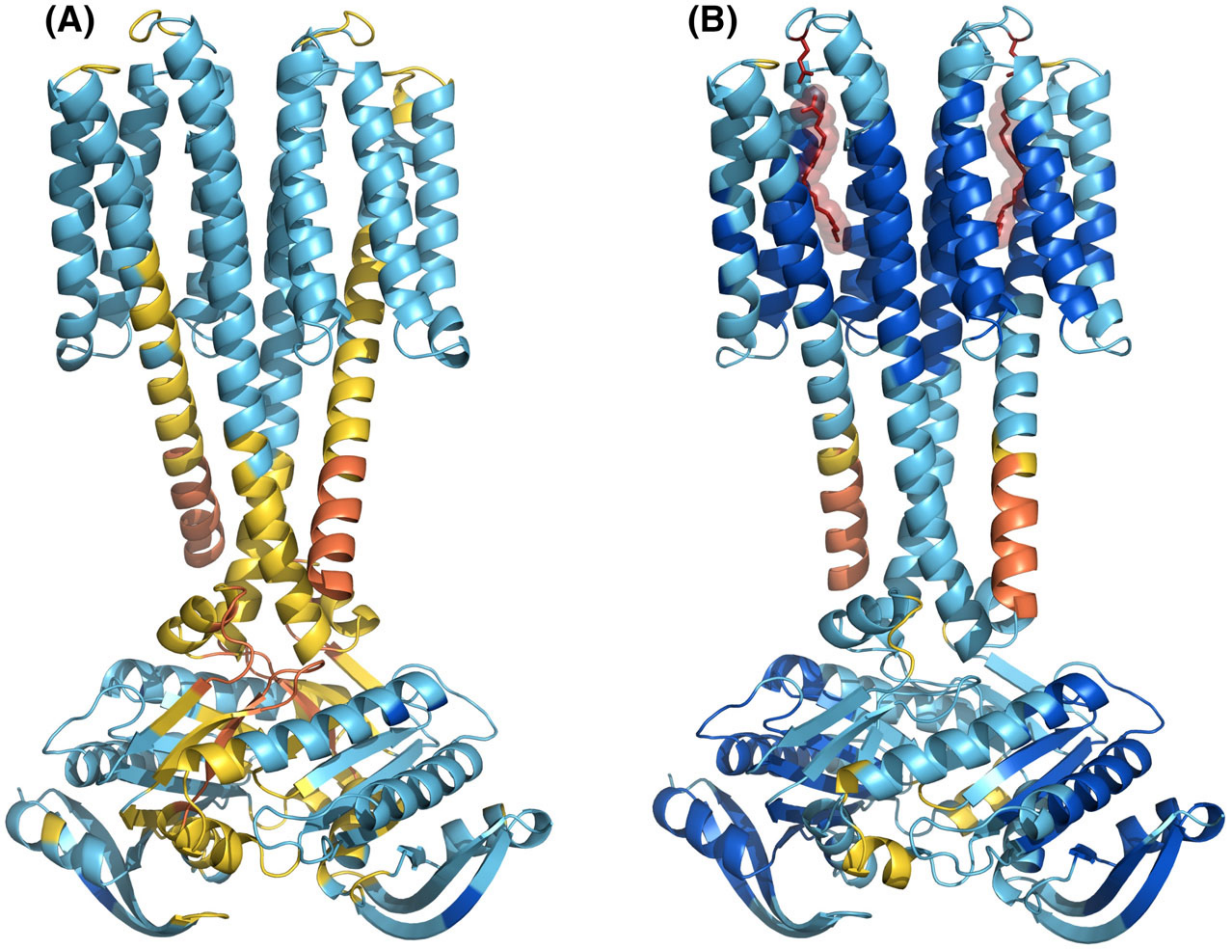
Models of Rv1625c computed by alphafold3. The models were obtained from the alphafold server at https://alphafoldserver.com/ by entering two copies of the Rv1625c sequence with and without two molecules of oleic acid as ligands and are colored by pIDDT values as on the alphafold server, with decreasing confidence from dark blue to orange. The N‐terminal 21 residues and C‐terminal 17 residues, which are modeled with low confidence as unstructured, are not shown. (A) The apo‐form of the Rv1625c dimer. (B) The holo‐form of the dimer. The two molecules of oleic acid and the side‐chains of Q174, which is the only residue forming polar contacts with the ligand, are shown in red color.

While the binding site of oleic acid in the Rv1625c receptor domain remains to be established experimentally, models obtained with alphafold3 for Rv1625c dimers with and without oleic acid (Fig. 6) support our previous proposal that the lipid ligands bind into a deep pocket formed by the transmembrane α‐helices [2]. It is noteworthy that apo‐ and holo‐models computed by alphafold are within 1.5 Å root‐mean‐square‐deviation of each other in Cα‐carbon positions, but the model confidence, as measured by pIDDT values, improves markedly upon the addition of oleic acid (Fig. 6, panels A vs. B). Each subunit binds a molecule of oleic acid into a deep pocket perpendicular to the plane of the membrane, with the carboxyl group of the ligand near the extracellular surface of the protein. The pocket is capped by a glutamine residue (Q174), which projects from the extracellular loop connecting helices 5 and 6 of the membrane domain and provides the only polar contact to the ligand. This residue is highly conserved in more than 170 orthologs of Rv1625c from other Actinobacteria [9]. The binding of two oleic acids into the Rv1625c catalytic dimer supports the strong cooperativity of inhibition as indicated by the Hill coefficient (see above).

Second, and surprisingly, oleic acid inhibitsRv1625c in the presence of Mg^2+^‐ATP, but not Mn^2+^‐ATP. In the past, Mn^2+^ or Mg^2+^ were used almost interchangeably as divalent cations for ATP in AC assays. Generally, Mn^2+^ greatly increases the activity of bacterial and mammalian ACs. The differing roles of Mg^2+^ and Mn^2+^ as cations were investigated biochemically in the past, and two binding sites for divalent cations were invoked [26, 27, 28, 29, 30, 34, 35]. Later, structural work identified two binding sites for divalent cations in the active center binding either Mg^2+^, Mn^2+^ or Zn^2+^ [36]. It is highly probable that cAMP biosynthesis by Rv1625 similarly proceeds via a two‐metal‐ion catalysis. Mn^2+^ appears to stabilize the enzymatic transition state more efficiently than does Mg^2+^ [36]. Our findings may also be consistent with the existence of a third binding site for a divalent cation that is directly involved in intramolecular signal transduction. We assume that this binding site for a divalent cation is positioned along the α‐helix that links the membrane receptor domain with the catalytic AC dimer (see Fig. 1) because Mn^2+^ appears to block intramolecular signaling. The reasons for the discrimination between Mg^2+^and Mn^2+^ in intramolecular signal transduction are currently inexplicable. Mg^2+^and Mn^2+^ differ in the spin states and in the number of potential coordination sites.

Third, obviously, the direct lipid regulation of mammalian mACs originated in the bacterial world and was retained in the mammalian AC lineage. This bolsters the close evolutionary association between bacterial ACs like Rv1625c and their mammalian congeners. Apparently, lipid signaling has emerged early in evolution. It uses an essential primordial cell constituent, a fatty acid. Lipid signaling may thus resemble other signaling systems that use basic components required for assembly of a primordial cell, such as amino acids or nucleotides, e.g., in GABA‐ergic [37, 38], glycine‐ergic [39, 40] or purinergic signaling [41]. Lipid signaling has persisted over billions of years of evolution. Only later in evolution, the regulatory GPCR/Gsα system has evolved in eukaryotes [42] and now mammalian mACs are receivers of the GPCR/Gsα signaling system. In mammals, lipid signaling has been retained as a tonic signal that modulates the indirect mAC stimulation by the GPCR system.

Fourth, the data establish a functional symmetry between purine nucleotidyl cyclases [6, 43, 44]. Both cyclases exist as membrane‐bound (mAC, mGC) and as soluble, cytosolic proteins. In mGCs, the extended N‐terminal domain (~ 440 aa) of the single‐pass transmembrane domain is an atrial natriuretic peptide receptor, whereas the soluble GCs are activated by gaseous nitric oxide. The membrane domains of class III AC isoforms, which possess only short, are lipid receptors, and the ligands bind into the membrane space. The regulation of the soluble AC (isoform) has not been finally elucidated [45, 46]. One may cautiously predict the existence of an endogenous cytosolic constituent regulating the soluble mammalian AC (AC isoform 10).

Lastly, our studies were restricted to the identification of a regulator/modulator of the membrane domain of Rv1625c. Therefore, we cannot and do not wish to discuss potential physiological or pathological implications. Each of the 12 sequenced mycobacteria strains possesses at least 4 (*M. leprae*) and up to 31 (*M. marinum*) class III AC isoforms. So far, convincing biochemical data of distinct physiological functions are missing. In general, mycobacteria are soil bacteria and only a few strains are human or animal pathogenic. Therefore, a direct and decisive role in pathogenesis appears questionable.

Another unresolved, yet important question is the source of lipid ligands in the mycobacterial environment. In a soil habitat, lipids could, probably will, be used in chemical interactions in bacterial communities. In an infected patient, signaling interactions between pathogen and host via lipids is a real possibility, albeit as yet unproven.

## Materials and methods

### Reagents and materials

ATP, creatine kinase, creatine phosphate, and lipids were from Merck‐Sigma. Stock solutions were prepared in DMSO and kept under nitrogen. The DMSO concentrations in assays maximally were 1%, a concentration without biochemical effects.

### Plasmids and protein expression

pQE30 plasmids for the Rv1625c AC holoenzyme and the C‐ to N‐terminal‐linked mycobacterial Rv1625c AC‐CAT‐_(204–443)2_ dimer (linker sequence TRAAGGPPAAGGLE) were available in the laboratory and expressed and purified as described [1]. The soluble construct was purified via its N‐terminal MRGSH_6_GS‐tag using NTA (Qiagen, Hilden, Germany) as described [1].

### Adenylyl cyclase assay

AC activities were measured in a volume of 10 μL for 15 min at 37 °C using 2 mm ATP, 2 mm MgCl_2_, or 2 mm MnCl_2_ where indicated, 3 mm creatine phosphate, 60 μg·mL^−1^ creatine kinase, and 50 mm MOPS pH 7.5. The cAMP assay kit from Cisbio (Codolet, France) was used for cAMP determination according to the supplier's instructions. For each assay, a cAMP standard curve was established. Two technical repeats were carried out for each data point.

### Data handling and analysis

Experimental results were evaluated using graphpad prism version 10.4.1. Graphs were generated by graphpad and assembled in powerpoint.

## Author contributions

AS planned and performed experiments, analyzed data; ML contributed to data curation, validation, and visualization; ANL contributed to structural modeling and interpretation; JES contributed to conceptualization, formal analysis, supervision, and wrote the paper.

## Conflict of interest

The authors declare no conflict of interest.

## Peer review

The peer review history for this article is available at https://www.webofscience.com/api/gateway/wos/peer‐review/10.1111/febs.70148.

## Data Availability

The data that support the findings of this study are available from the corresponding author (joachim.schultz@tuebingen.mpg.de) upon reasonable request.
